# Variability in Self-Reported Preoperative Pain Between Clinic Visits and Day of Surgery

**DOI:** 10.7759/cureus.90586

**Published:** 2025-08-20

**Authors:** David E Komatsu, Sunitha M Singh, Susannah Oster, James Espeleta, Maheen Khan, Harry Divaris, Sarah Landman, Samuel Stanley, Elliott Bennett-Guerrero, Martin Kaczocha

**Affiliations:** 1 Orthopaedics and Rehabilitation, Stony Brook University, Stony Brook, USA; 2 Anesthesiology, Stony Brook University, Stony Brook, USA

**Keywords:** numerical rating scale, pain, preoperative, spinal fusion, total hip arthroplasty, total knee arthroplasty

## Abstract

Background: Preoperative pain is routinely reported in the clinical literature and is benchmarked against numerous clinical parameters, necessitating that baseline preoperative pain is precisely defined and exhibits minimal variability. The goal of this study was to determine whether there is variability in pain scores obtained on the day of surgery (DOS) compared to preoperative clinic visits.

Methods: We conducted a retrospective analysis of total knee arthroplasty (TKA), total hip arthroplasty (THA), and spinal fusion (SF). Self-reported 0-10 numeric rating scale (NRS) pain scores were compared between clinic visits and DOS in 1,567 patients. The primary intent was to determine if there were differences between clinical and DOS NRS scores. Secondary analyses were performed by stratifying the results by procedure, gender, and time of clinic visit. Additionally, we performed a qualitative literature review to assess how the timing of preoperative pain assessments is reported.

Results: Our primary analysis found patients undergoing painful procedures reported significantly greater pain in the clinic compared to DOS, with a median difference of 3 and an interquartile range (IQR) of 6 (p < 0.0001). For our secondary analyses, similar differences between clinic and DOS were found for TKA (3; IQR 7; p < 0.0001), THA (3; IQR 7; p < 0.0001), and SF (2; IQR 5; p < 0.0001). Stratification by gender revealed comparable reductions (p = 0.6985). Finally, these discrepancies were consistent across preoperative clinic visits ranging from 1-90 days prior to surgery compared to DOS (p = 0.1944). Our qualitative literature review revealed that the timing of preoperative pain acquisition is rarely reported.

Conclusions: Our results indicate that the degree of patient-reported pain differs drastically between clinic visits and DOS and identify a potential source of interstudy variability, highlighting the importance of defining the timing and setting of preoperative pain assessments.

## Introduction

Over 300 million surgeries are performed annually worldwide, with more than 80% accompanied by acute postsurgical pain [1,2]. A large proportion of these surgeries are conducted for orthopaedic issues, such as total knee arthroplasty (TKA), total hip arthroplasty (THA), and spinal fusion. Importantly, the annual number of TKA and THA surgeries is rapidly rising, with TKA and THA surgery projected to reach 3,500,000 and 572,000 cases per year by 2030, respectively [3].

Management of postoperative pain continues to be a major challenge, and inadequately controlled pain negatively affects patient satisfaction and quality of life, leads to increased utilization of opioids, and is a risk factor for the development of chronic postsurgical pain [4,5]. Consequently, significant effort has been dedicated to characterizing preoperative factors that influence postoperative pain, with an extensive body of work demonstrating that patients presenting with higher levels of preoperative pain report greater postoperative pain [4,6-9]. Other factors associated with higher levels of postoperative pain include depression [10], sleep disorders [11], female sex [12], and elevated body mass index [13]. Given the association between the magnitude of pre- and postoperative pain and the potential influence of preoperative pain upon additional perioperative parameters, it is imperative that baseline presurgical pain is well-defined and factors that induce variability in patient-reported pain scores are identified. The precise definition of baseline pain and strategies to limit variability in patient-reported scores are also critically important parameters in chronic pain research and clinical trial design [14,15].

During the course of a prospective study assessing preoperative and postoperative pain in patients undergoing TKA, we noted differences in preoperative pain scores obtained at the clinic and on the day of surgery (DOS), suggesting that the location and/or timing between pain scores taken at the clinic and DOS may influence patient-reported pain. We subsequently performed a qualitative review of recent studies reporting preoperative pain in patients undergoing TKA, THA, and spinal fusion, procedures that are predominantly performed on patients with underlying pain, the results of which indicate that the location and time interval prior to surgery are not routinely reported. Based on these findings, we conducted a retrospective study comparing patient-reported pain scores obtained during preoperative clinic visits and the DOS across several procedures associated with preoperative pain. Therefore, this study has two objectives: (i) to complete a retrospective analysis to ascertain if there are significant differences in self-reported pain scores obtained at the clinic and the DOS, and (ii) to perform a qualitative literature review to determine how preoperative pain scores are commonly reported. The completion of these objectives will facilitate a better understanding of a potential source of variability in preoperative pain reporting.

## Materials and methods

A retrospective, single-arm study was conducted at Stony Brook University Hospital, New York, United States, of selected patients who underwent surgery between January 1, 2017, to December 31, 2021. The study was approved by the Stony Brook University Institutional Review Board on June 21, 2022, under exemption category 45 CFR 46.104.d.4iii., with a waiver of informed consent (IRB2022-00300).

Eligibility criteria

Adult (≥18 years) patients undergoing elective TKA, THA, or spinal fusion were included. Exclusion criteria included surgical indications resulting from trauma, revision arthroplasty, multiple surgeries at the time of index procedure, not having both clinic and date of surgery pain scores, cancer or fracture indication for TKA, THA, or fusion, >90 days between most recent clinic visit and surgery, pregnancy, and surgery postponed >14 days.

Inclusion criteria for the control group were adult (≥18 years) patients undergoing elective thyroidectomy, parathyroidectomy, or nephrectomy, who were unlikely to have preoperative pain.

Data collection

For the primary analysis, which assessed variability in preoperative pain scores in patients with pain preoperatively, data were collected from patients undergoing procedures generally associated with preoperative pain. Data were collected from the electronic medical records (EMRs) onto hard copy case report forms (CRFs). The data from the CRFs were collected and managed using REDCap electronic data capture tools hosted at Stony Brook University.

Data collected included: age at surgery, gender, ethnicity, race, height, weight, body mass index (BMI), American Society of Anesthesiologists (ASA) score, planned surgical procedure and date, indication for surgery, preoperative diagnosis, preoperative medications, actual surgical procedure and date, numerical rating scale (NRS) pain scores from the clinic visit closest to the surgical date, and NRS scores on the DOS, prior to the administration of any analgesics.

NRS is a standard research tool that is not under any copyright protections. Gender, ethnicity, and race were all self-reported and were collected and reported to characterize the study purpose for comparison to other studies, as well as to facilitate study generalizability. A data dictionary was developed to ensure consistent data abstraction from discrete locations within the EMR. Data abstractors were trained in data collection in several sessions and did not collect data until consistency in collection was demonstrated. The data abstractors were then randomly assigned to collect data from the same number of subjects per surgical indication. To ensure consistency, CRFs were spot checked by the senior and corresponding authors. Any discrepancies in values to record were adjudicated in regular group discussions to reach consensus.

In order to confirm the internal validity of self-reported pain scores at the two hospital sites (clinic visit vs. hospital on DOS), we also collected data from patients who were unlikely to have pain preoperatively. If we could demonstrate that these patients had little to no pain at these two sites, it would support the validity of any observed pain scores in the primary analysis.

Participants

**Figure 1:**
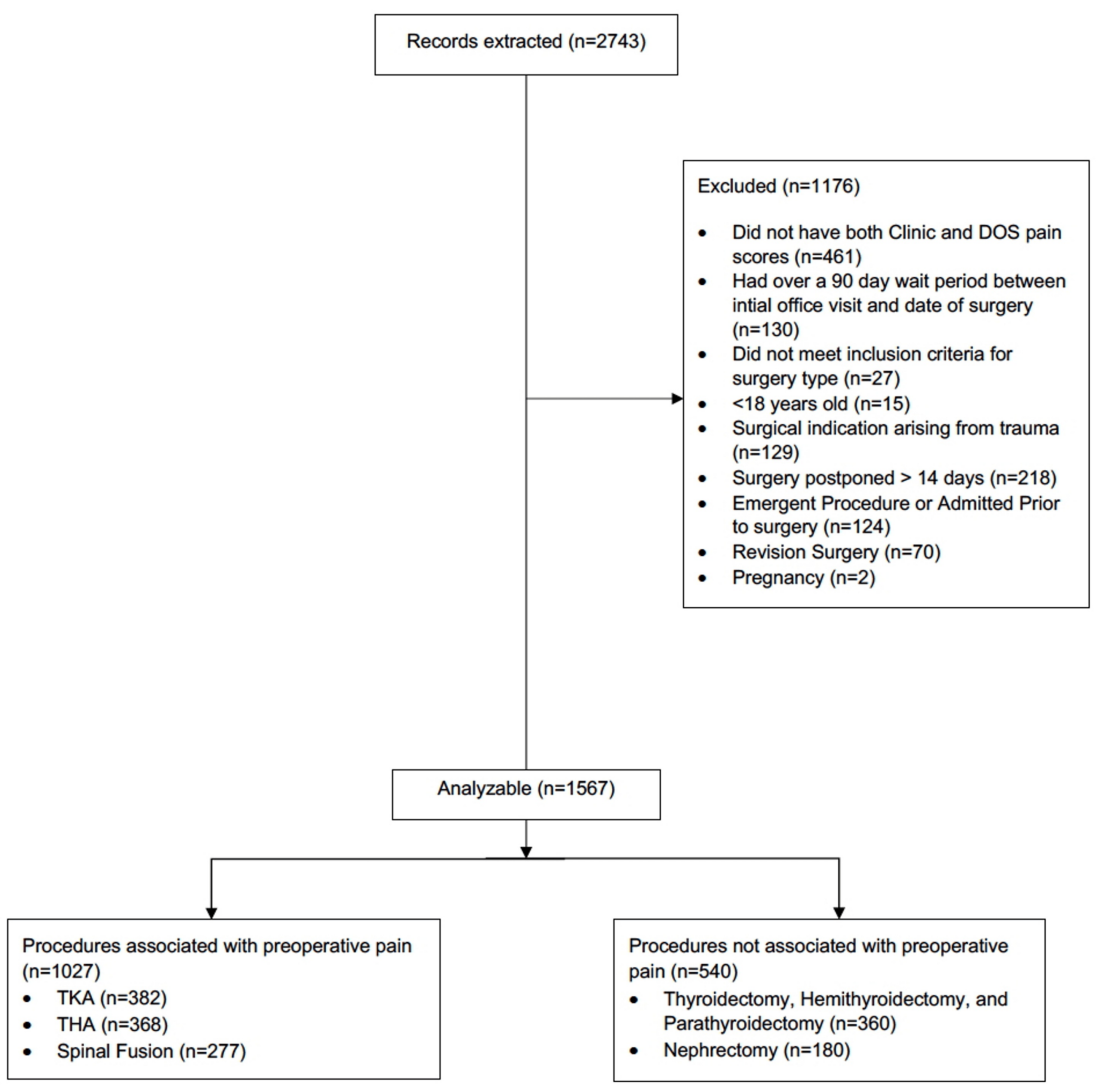
Flow diagram of the subject selection DOS: day of surgery; TKA: total knee arthroplasty; THA: total hip arthroplasty

We screened 2,743 electronic medical records with a total of 1,567 patients meeting the inclusion criteria (Figure 1).

Statistical analysis

No power analysis was conducted for the study, as the sample size is an order of magnitude larger than in similar papers. Median and interquartile range (IQR) are reported for all ordinal variables. Shapiro-Wilk tests for normality were performed on the distribution of pain scores, which were not found to be normally distributed; as such, non-parametric tests were used to analyze all data. The primary objective was to test the null hypothesis that there was no difference in NRS pain scores between clinic and date of surgery for patients likely to have preoperative pain. The primary analysis was performed using a Wilcoxon signed-rank test to compare the differences in pain scores between clinic visits and DOS for patients who had both pain scores and had a procedure associated with preoperative pain. Three Wilcoxon signed-rank tests were performed on the three separate procedures in this group (TKA, THA, spinal fusion). A Kruskal-Wallis test was subsequently performed to determine if the differences in NRS pain scores between DOS and clinic visits for the painful procedures (THA, TKA, spinal fusion) differed between these procedures. Significant pairwise differences were then identified using a Dunn’s Test.

In addition, a Wilcoxon Rank-Sum test was used to test if there was a significant difference between male and female NRS pain differences from the preoperative clinic visit to DOS in these patients. After stratifying by gender, two separate Wilcoxon signed-rank tests were performed to test if the differences in NRS pain scores from preoperative clinic visit to DOS were significant in the male and female patients who underwent painful procedures. Finally, a Kruskal-Wallis test was used to determine if time from clinic visit to DOS had an effect on pain differences in this group; a categorical time variable was created by splitting the patients into preoperative clinic visits ranging from 1-14 days, 15-28 days, 29-42 days, 43-56 days, 57-70 days, and 71-90 days prior to surgery. An alpha level of 0.05 was used to determine statistical significance. All analyses were performed in SAS 9.4 (SAS Institute Inc., Cary, North Carolina, United States).

In order to confirm the internal validity of self-reported pain scores at the two hospital sites (clinic visit vs hospital on DOS), we sought to show an expected higher pain level in our primary analysis population, compared with patients not expected to have pain preoperatively. A Wilcoxon signed-rank test was used to test if there was an expected difference between patients in these two groups.

## Results

Patient reported differences in pain ratings at the clinic and DOS

**Figure 2:**
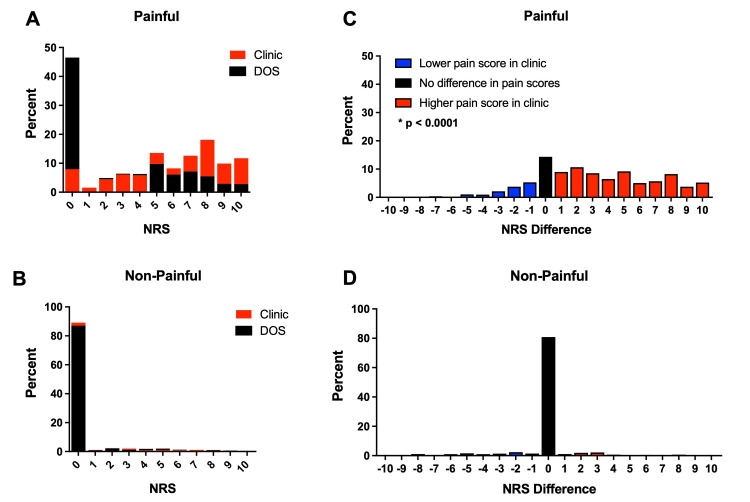
Distribution of Pain Scores at Clinic and DOS. Distribution/Histograms of NRS scores for (A) Painful procedures obtained at the clinic (red) and DOS (black) and (B) Non-Painful procedures obtained at the clinic (red) and DOS (black); (C) Distribution of differences in pain scores for painful procedures. Differences in preoperative pain scores obtained in the clinic and DOS are presented as NRS scores in the clinic – DOS. Red bars indicate a higher pain score in clinic compared to DOS, blue bars a lower pain score in clinic compared to DOS, and black more indicate no change. The median difference in pain scores was 3, with an IQR of 6 (Wilcoxon Signed-Rank test; p < 0.0001); (D) Distribution of differences in pain scores for non-painful procedures. The median difference in pain scores was 0, with an IQR of 0 (Wilcoxon Signed-Rank test; p = 0.2176). DOS: day of surgery; IQR: interquartile range; NRS: numerical rating scale

The primary objective of this retrospective study was to compare self-reported pain scores obtained at the clinic and DOS for procedures associated with preoperative pain (Painful), which consisted of TKA (n=382), THA (n=368), and spinal fusion (n=277). Patient characteristics are presented in Table 1. The numeric rating scale (NRS) obtained at the clinic spanned the entire NRS range (0-10), with high pain scores (>5) predominant in this cohort (Figure 2A). Strikingly, our analysis of the DOS NRS scores revealed a significant shift, with the largest number of patients reporting a NRS score of 0 (Figure 2C). We then compared the median difference in pain scores between the clinic and DOS and found it to be 3 points higher with an IQR of 6 (Figure 2E), indicating that patients reported significantly more pain at the clinic than on the DOS.

**Table 1:** Characteristics of subjects IQR: interquartile range

Characteristics	Painful (N=1032)	Non-Painful (N=542)	Total (N=1574)
Days from clinic visit to DOS, median (IQR)	40.5 (26.0, 61.0)	33.0 (18.0, 48.0)	39.0 (23.0, 57.0)
Age at Surgery (years), median (IQR)	65.0 (57.0, 73.0)	57.0 (47.0, 67.0)	63.0 (54.0, 72.0)
BMI (kg/m^2^), median (IQR)	30.7 (27.0, 35.2)	29.1 (25.3, 33.6)	30.3 (26.4, 34.7)
Gender, n (%)			
Male	461 (44.7%)	202 (37.3%)	663 (42.1%)
Female	571 (55.3%)	340 (62.7%)	911 (57.9%)
Ethnicity, n (%)			
Other	42 (4.1%)	52 (9.6%)	94 (6.0%)
Unknown	7 (0.7%)	3 (0.6%)	10 (0.6%)
Hispanic	23 (2.2%)	29 (5.4%)	52 (3.3%)
Non-Hispanic	960 (93.0%)	458 (84.5%)	1418 (90.1%)
Race, n (%)			
Asian	9 (0.9%)	13 (2.4%)	22 (1.4%)
Black	46 (4.5%)	50 (9.2%)	96 (6.1%)
Indian	4 (0.4%)	1 (0.2%)	5 (0.3%)
Other	38 (3.7%)	53 (9.8%)	91 (5.8%)
Unknown	1 (0.1%)	7 (1.3%)	8 (0.5%)
White	934 (90.5%)	418 (77.1%)	1352 (85.9%)

To establish internal validity, we also evaluated self-reported pain scores for patients undergoing procedures not associated with preoperative pain (Non-Painful). This consisted of thyroidectomy, hemithyroidectomy, and parathyroidectomy (n=360), and nephrectomy (n=180). As expected, the vast majority of these patients reported NRS scores of 0 at the clinic and DOS (Figure 2B). Moreover, the NRS scores between the clinic and DOS did not significantly differ (Figure 2D), supporting the internal validity of the pain assessments at both time points.

Influence of surgical procedure, gender, and time interval prior to surgery upon differences in self-reported pain

**Figure 3:**
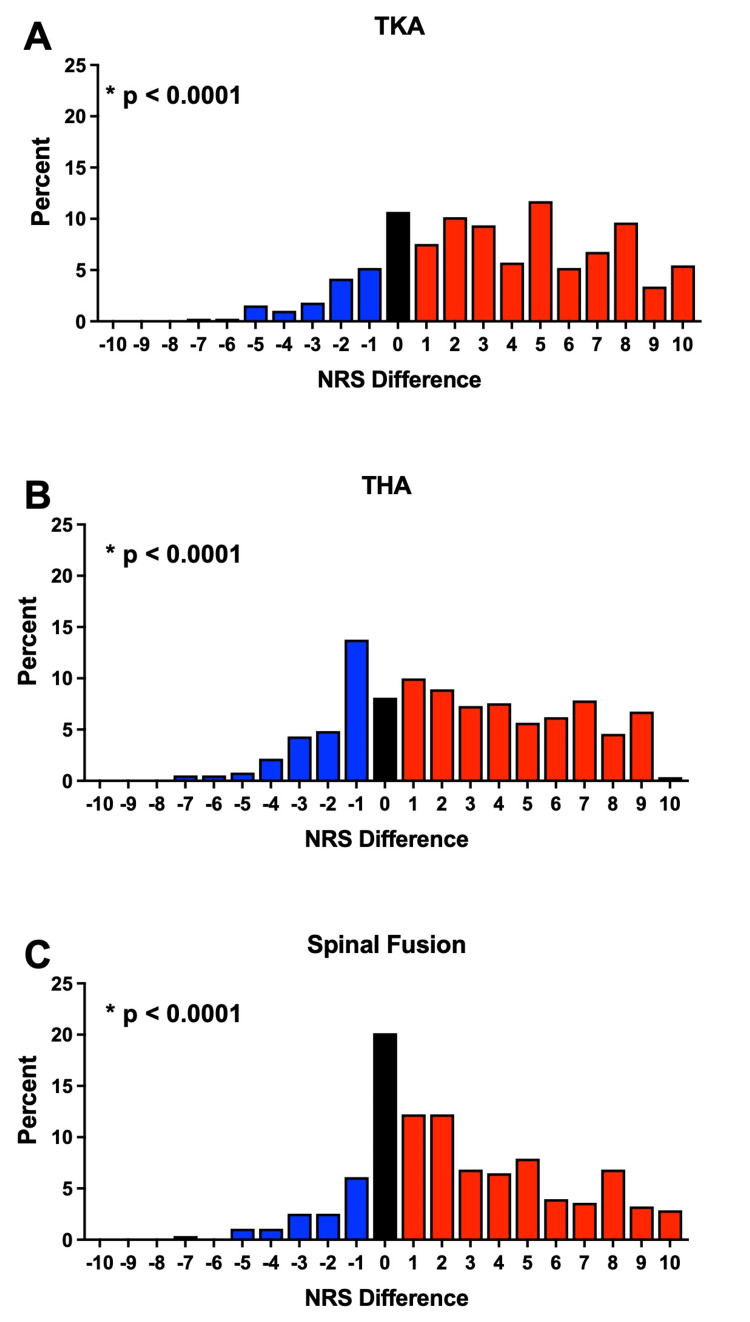
Differences in pain scores between the clinic and DOS stratified by procedure. Differences in preoperative pain scores obtained in the clinic and DOS are presented as NRS scores in the clinic – DOS. Red bars indicate a higher pain score in clinic compared to DOS, blue bars a lower pain score in clinic compared to DOS, and black more indicates no change. (A) The median difference in pain scores for TKA between clinic and DOS was 3, with an IQR of 7 (Wilcoxon Signed-Rank test; p < 0.0001); (B) The median difference in pain scores for THA was 3, with an IQR of 7 (Wilcoxon Signed-Rank test; p < 0.0001). (C) The median difference in pain scores for spinal fusion was 2, with an IQR of 5 (Wilcoxon Signed-Rank test; p < 0.0001). Comparison between procedures found a significant difference in the differences in pain scores between procedures (Kruskal-Wallis; p=0.0023), with differences found between spinal fusion and TKA (Dunn’s; p=0.0013) and THA (Dunn’s; p=0.0035), respectively. DOS: day of surgery; IQR: interquartile range; TKA: total knee arthroplasty; THS: total hip arthroplasty; NRS: numerical rating scale

**Figure 4:**
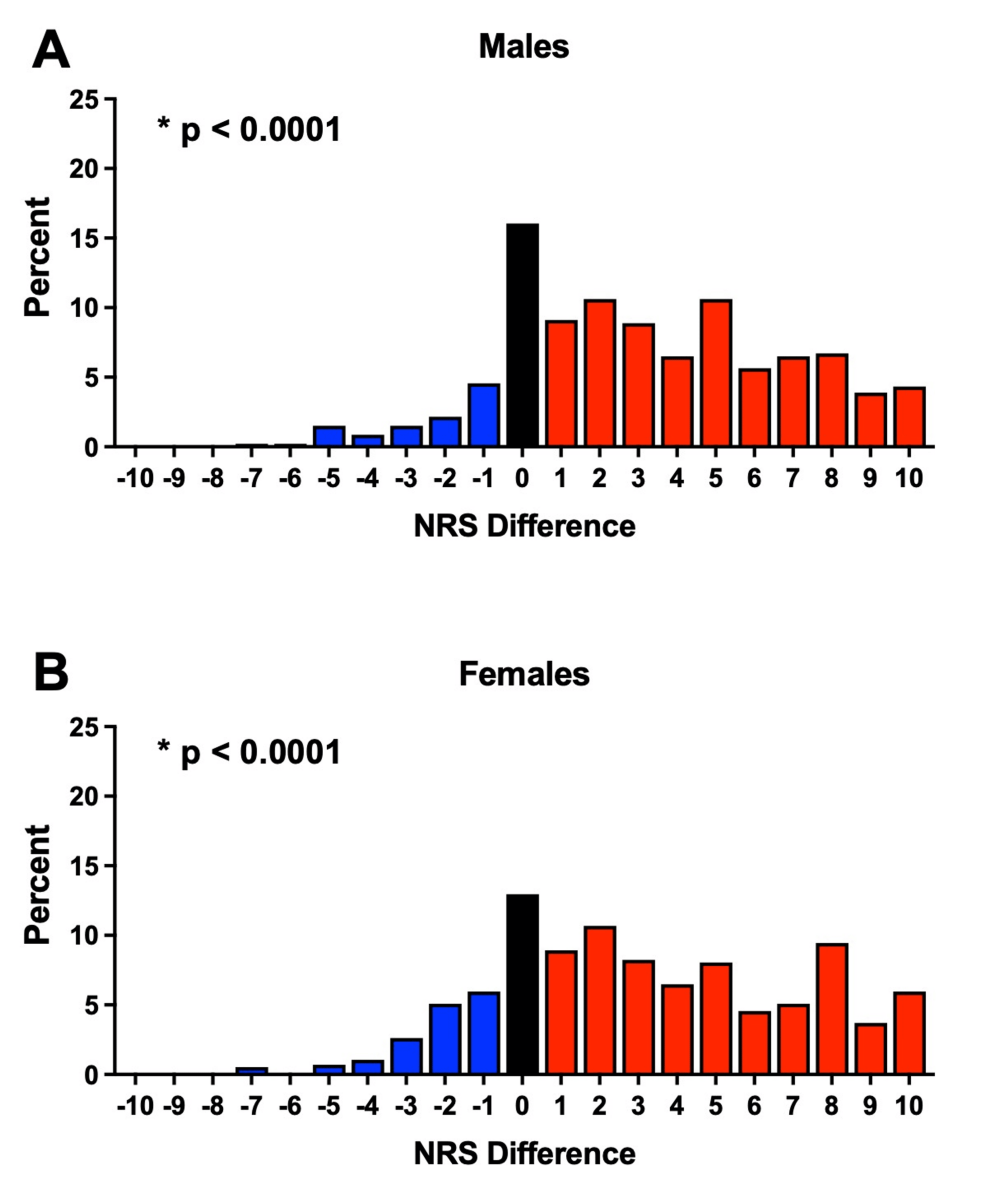
Differences in Pain Scores by Gender. Differences in preoperative pain scores obtained in the clinic and DOS are presented as NRS scores in the clinic – DOS. Red bars indicate a higher pain score in clinic compared to DOS, blue bars a lower pain score in clinic compared to DOS, and black more indicates no change. (A) The median difference in pain scores for painful procedures in males between clinic and DOS was 3 with and IQR of 6 (Wilcoxon Signed-Rank test; p < 0.0001). (B) The median difference in pain scores for painful procedures in females between clinic and DOS was 3 with and IQR of 6 (Wilcoxon Signed-Rank test; p < 0.0001). The median of pain differences did not differ between male and female subjects (Wilcoxon rank sum test; p = 0.6985). DOS: day of surgery; IQR: interquartile range; NRS: numerical rating scale

**Figure 5:**
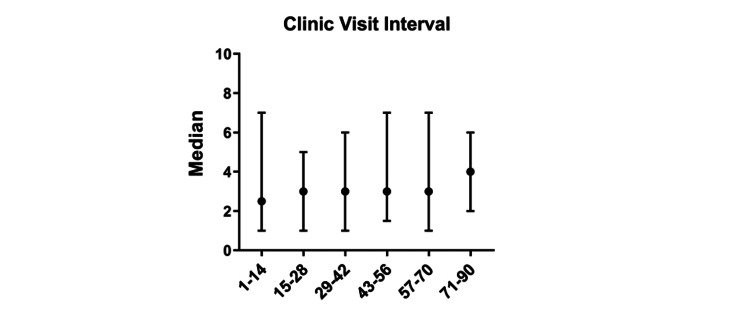
Differences in Pain Scores by Time. Absolute pain differences between clinic and DOS (Median +/- IQR) stratified by time interval (days) of clinic visit prior to DOS. No significant differences were identified (Kruskal-Wallis test; p = 0.1944). DOS: day of surgery; IQR: interquartile range

We subsequently examined whether the shift in NRS scores between the clinic and DOS seen in the Painful cohort was common for all three procedures. Indeed, stratification by TKA, THA, and spinal fusion demonstrated that a significant shift in pain scores (TKA: p<0.001; THA: p<0.0001; spinal fusion: p<0.0001) occurred irrespective of the surgical indication (Figure 3). Next, we compared the differences in clinic and DOS NRS pain scores between these surgical indications and found that there was a significant difference between the three procedures (p=0.0023). Post-hoc testing revealed that differences in preoperative pain between the clinic and DOS for the spinal fusion patients were significantly different from both TKA (p=0.0013) and THA (p=0.0035) patients. No differences were seen between TKA and THA patients (p=0.760). When stratified by gender, male and female subjects were similar to the total Painful cohort in that each gender had significant differences in pain scores (Male: p < 0.0001; Female: p < 0.0001). Moreover, the differences in the pain scores from clinic to DOS did not differ between male and female subjects (p=0.6985), signifying that the reductions in self-reported pain between the clinic and DOS were independent of gender (Figure 4). Lastly, we sought to determine whether the time interval between pain assessment at the clinic and DOS influenced the results. The magnitude of the median pain difference for pain scores obtained at the clinic from one to ninety days prior to the DOS did not significantly differ (p=0.1944) (Figure 5). Collectively, our results suggest that the assessment of preoperative pain in the clinic compared to DOS has a significant influence upon patient-reported pain scores for surgical procedures associated with preoperative pain.

Qualitative literature review

We performed a qualitative review to determine the general reporting standards for the timing of preoperative pain assessments. This review was conducted by performing PubMed searches using the keywords “VAS”, “NRS”, or “pain”, and “total knee arthroplasty”, “total hip arthroplasty”, or “spinal fusion” to identify studies on TKA, THA, and spinal fusion, respectively. Other inclusion criteria were: English language, primary research studies, studies with abstracts, and those published between January 2022 and February 2023. The abstracts for these studies were read in reverse chronological order (i.e., newest to oldest), and those that reported preoperative pain scores were included in the analysis. Once 25 studies had been identified for inclusion for each surgical procedure, no further studies were evaluated.

The results of the review are presented in Tables 2-4. For TKA, 24 studies were included [16-39], and of these, only one study by Meena et al. reported on the timing of preoperative pain score collection [16]. Specifically, they stated that “Preoperatively, patients’ condition was evaluated four weeks before the surgery by questionnaire rather than immediately before surgery”. The remaining 23 TKA studies did not report the timing of preoperative pain assessments. For THA, 20 studies were included [21,29,40-56], and only the study by Singh et al. reported timing of pain score collection, stating that “Patients were preoperatively registered for an electronic patient engagement application by clinical care coordinators at the time of surgical scheduling. This application was used to collect VAS pain scores and quantity of opioid consumption daily for seven days before surgery through the first 30 postoperative days" [44]. Finally, for spinal fusion, 25 studies were included [57-81], and none of them explicitly reported the timing of preoperative pain score assessments.

**Table 2:** Summary of included studies for total knee arthroplasty VAS: visual analog scale; KSS: Knee Society Score; PCS: Pain Catastrophizing Scale; HSS: Hospital for Special Surgery; ROM: range of motion; OKS: Oxford Knee Score; WOMAC: Western Ontario and McMaster Universities Osteoarthritis Index; TKA: total knee arthroplasty; PROMS: patient-reported outcome measures; KOOS-JR: Knee Injury and Osteoarthritis Outcome Score, Joint Replacement; POD: postoperative day; VR-12: Veterans RAND 12 Item Health Survey

Authors	Year	Study Type	Time Specified (Y/N)	Description of Collection Time
Lu et al. [19]	2023	Prospective observational	N	After receiving an explanation of the research program, each patient was asked to complete a questionnaire booklet containing the VAS for pain severity.
Kyriakidis et al. [20]	2023	Prospective observational	N	Preoperative and postoperative evaluation included knee-related clinical and functional assessment based on objective and subjective scores, including the knee flexion, the extension lag, the KSS clinical and functional, and the VAS for pain at 12 and 24 months.
Fard et al. [21]	2022	Prospective randomized double blind	N	The outcome measure included a five-step evaluation of VAS (preoperative, 12 hours, 24 hours, 48 hours, and 72 hours after the operation).
Qiao et al. [22]	2022	Retrospective	N	The following outcomes were compared for changes between preoperative and last follow-up results: erythrocyte sedimentation rate, C-reactive protein, VAS scores, HSS scores, knee ROM, and infection cure rates.
Gao et al. [23]	2022	Prospective randomized double blind	N	A physiotherapist blinded to group allocation recorded self-reported postoperative VAS scores. The patients rated postoperative pain with movement on PODs 1–2, at discharge, at 1 month, and 1 year after surgery.
Oncel et al. [24]	2023	Prospective observational	N	The International Consultation on Incontinence Question-Short Form (ICIQ-SF), Overactive Bladder-Validated 8 (OAB-V8), VAS, and OKS forms were completed pre-operatively and at the 6th post-operative month.
Zhang and Nie [25]	2022	Retrospective	N	Besides, preoperative and postoperative knee joint HSS scores, knee joint pain assessed by VAS, knee joint ROM, and bone metabolism parameters (osteocalcin, total N-terminal propeptide of type I procollagen, and β-isomerized C-terminal telopeptides) were recorded.
Heller et al. [26]	2022	Prospective randomized	N	Pain was measured using a numeric VAS 1–10 on the preoperative visit, and on POD1 prior to manipulation, with active (patient-driven) movement and with passive (physician-elicited) movement.
Meena et al. [16]	2023	Prospective matched control	Y	Preoperatively, patients’ condition was evaluated 4 weeks before the surgery by questionnaire rather than immediately before surgery.
Ao et al. [17]	2022	Prospective observational	N	The pain was measured by the VAS pre- and postoperatively.
Geng et al. [27]	2022	Prospective randomized double blind	N	The VAS scores in rest and in motion were evaluated on POD 1, POD 3, and POD 30. HSS scores were evaluated preoperatively and on POD 3 and POD 30.
Naoum et al. [28]	2022	Prospective observational	N	Patients’ demographics, the preoperative range of flexion, the flexion contracture, as well as the preoperative VAS score for pain, the degree of coronal plane deformity (varus-valgus), the Kellgren and Lawrence radiological degree of osteoarthritis (K-L Classification), and the Beguin and Locker classification of intraoperative degree of osteoarthritis (B-L Classification) were prospectively recorded.
Harris et al. [29]	2022	Retrospective	N	The preoperative PROMs were the Oxford Hip Score or OKS, the EQ-5D Utility Index, and the EQ VAS for overall health.
Liao and Xu [30]	2022	Retrospective	N	VAS scale was used to evaluate the pain level at rest and during exercise before surgery, at 6 hours, 24 hours, 48 hours, and 72 hours after surgery.
Meena et al. [31]	2023	Retrospective	N	Sports participation and sports preference, OKS, Tegner Activity Level, and Visual Analogue Scale (VAS) for pain were recorded pre- and postoperatively at 6 months, 1 year, 2 years, and 5 years.
Kenanidis et al. [18]	2023	Prospective matched control	N	Complications, VAS score, and OKS were assessed preoperatively and for six postoperative months.
Haffar et al. [32]	2022	Prospective randomized double blind	N	Outcomes included VAS pain and numeric rating scale sleep scores (collected on POD 0, 1, 2, 7, 14, 42), and cumulative postoperative opioid use (42 days).
LeBrun et al. [33]	2022	Retrospective	N	In this retrospective matched cohort study evaluating Medicare patients who underwent telerehab or conventional physiotherapy after TKA, we found that telerehab use was not associated with an increase in 90-day unplanned healthcare encounters, 120-day MUAs, or changes from baseline in the KOOS-JR, VAS, or VR-12.
Chang et al. [34]	2022	Prospective observational	N	Patients’ subjective perception of resting pain was estimated using the VAS score, which is widely used and determined by measuring the distance on a 10-cm line between the “no pain” anchor and mark made by the patient.
Chalidis et al. [35]	2021	Retrospective	N	Active knee ROM, VAS for pain, and KSS for pain and function were evaluated before and at least 6 months after neuroma excision, with a mean follow-up of 8 months (range: 6 to 11 mo).
Nielsen et al. [36]	2022	Prospective randomized double blind	N	Subject characteristics data included prior opioid treatment and PCS score, evaluating the level of or tendency to catastrophising thinking, along with baseline VAS (0–100 mm; 0 mm = no pain and 100 mm = worst possible pain) of pain at rest, at night, and at the 5 m walk test.
Kim et al. [37]	2021	Retrospective	N	Preoperative and postoperative PROMS (pain VAS and WOMAC scores were compared.
Xu et al. [38]	2021	Prospective randomized	N	Outcomes (joint function) were evaluated according to the KSS, VAS, WOMAC score, and ROM assessment at selected time points (preoperatively; 1 week; 1, 3, and 6 months; and 1 year after surgery).
Scott et al. [39]	2021	Prospective observational	N	Prior to and at 1 year following TKA, patients completed questionnaires including validated PROMs: the OKS; VAS Pain scores (0–100); and the EuroQol 5-dimension score (EQ-5D 3L).

**Table 3:** Summary of reviewed studies for total hip arthroplasty VAS: visual analog scale; OHS: Oxford Hip Score; THA: total hip arthroplasty; HHS: Harris Hip Score; WOMAC: Western Ontario and McMaster Universities Osteoarthritis Index; PROM: Patient-Reported Outcome Measures; NRS: Numerical Rating Scale; AOANJRR: Australian Orthopaedic Association National Joint Replacement Registry; SF: short form; LBP: lower back pain

Authors	Year	Study Type	Time Specified (Y/N)	Description of Collection Time
Shafiei et al. [40]	2022	Prospective randomized double blind	N	Pain was assessed using the VAS, with a scale from 0 (no pain) to 10 (most severe pain imaginable) at all visits.
Zimmerer et al. [41]	2022	Prospective observational	N	The pain level was evaluated using a VAS for pain (VAS 0 = no pain; VAS 10 = worst pain imaginable) and was recorded preoperatively and on each day of hospitalization for rest and motion.
Aggarwal et al. [42]	2022	Retrospective	N	Using preoperative data from a multisite registry, this study aimed to develop multivariable prediction models for the assessment of (i) patient-rated improvement, (ii) satisfaction, and (iii) OHS six months after THA.
Kobayashi et al. [43]	2023	Prospective observational	N	The VAS was used to assess knee pain severity before and at 3 months after THA.
Singh et al. [44]	2022	Retrospective	Y	As part of our institutional standard of care, patients were preoperatively registered for an electronic patient engagement application by clinical care coordinators at the time of surgical scheduling. This application was used to collect VAS pain scores and quantity of opioid consumption daily for seven days before surgery through the first 30 postoperative days.
Innocenti et al. [45]	2022	Prospective observational	N	Demographic and surgical characteristics, such as age, sex, and American Society of Anesthesiologists grade, were recorded preoperatively, along with the following questionnaires: modified OHS (mOHS), EuroQol five-dimension three-level health questionnaire (EQ-5D-3L), and a 100 mm VAS for pain during rest and during activity.
Fard et al. [21]	2022	Prospective randomized double blind	N	The outcome measure included a five-step evaluation of VAS (preoperative, 12 hours, 24 hours, 48 hours, and 72 hours after the operation.
Dubin and Westrich [46]	2022	Retrospective matched	N	The primary outcome measurements were PROMs, including HHS, WOMAC score, VR-12 score, UCLA activity score, and VAS pain score. All were collected preoperatively and at the latest follow-up visit.
Migliorini et al. [47]	2022	Systematic review	N	Data concerning the following endpoints at baseline were collected: Patient demographics: number of procedures, mean BMI and age, percentage of females; PROMs: VAS, OHS, WOMAC, HHS.
Simonsson et al. [48]	2022	Retrospective	N	PROM data collected from the SHAR via patient-filled questionnaires were analyzed. Variables were: Pain VAS, EQ VAS, and EQ-5D-SE (hip pain VAS, EuroQoL 5-dimensional health status questionnaire, Swedish experience-based.
Agerholm et al. [49]	2022	Retrospective	N	Data are based on patients' self-reported data from both pre- and post-operative surveys as well as medical data registered by healthcare staff.
Goyal et al. [50]	2022	Prospective observational	N	VAS for pain was recorded preoperatively and at 6, 12, 24, 48, and 72 h after the surgery and at 1st and 2nd year postoperatively.
Tottas et al. [51]	2022	Prospective observational	N	The levels of pain were recorded pre-operatively, at six hours, 12 hours, 24 hours, and 48 hours postoperatively based on the VAS/NRS score.
Harris et al. [29]	2022	Retrospective	N	Data were collected directly from patients who entered their responses electronically (via smartphone, tablet, or computer) through the AOANJRR online data collection system.
Morgan et al. [52]	2022	Retrospective	N	VAS scores were obtained pre-operatively and post-operatively.
Shang et al. [53]	2022	Retrospective	N	VAS, HHS, and Short-Form 36 (SF-36) values were recorded preoperatively, 3 and 12 months postoperatively, and at the last follow-up for patients in both groups.
Stoltny et al. [54]	2022	Retrospective	N	Clinical condition with the HHS, WOMAC scale, quality of life on the SF-12 scale, and pain VAS scale were assessed. Patients were evaluated in an average 7-year follow-up (range 4–9 years).
Kaseb et al. [55]	2022	Prospective randomized	N	Outcomes were measured before the intervention, one day, nine days, one month, and one year after the surgery.
Okizu et al. [56]	2022	Retrospective	N	The VAS score for LBP was collected by an interviewer, who asked patients whether they had any pain in the lumbar area, and the severity of the pain if they had LBP. Clinical scores included the HHS, OHS, and University of California, Los Angeles (UCLA) activity score, which were collected preoperatively and at 1 year postoperatively.

**Table 4:** Summary of reviewed studies for spinal fusion ODI: Oswestry Disability Index; VAS: visual analog scale; PROM: patient-reported outcome measure; LBP: lower back pain

Authors	Year	Study Type	Time Specified (Y/N)	Description of Collection Time
Hiranaka et al. [57]	2022	Prospective case control	N	Clinical outcomes were assessed according to the ODI and VAS for lower back pain, leg pain, and numbness preoperatively and at 1, 3, and 6 months, and 1 year postoperatively.
Nie et al. [58]	2023	Retrospective	N	VAS back/leg, and ODI were collected at preoperative and postoperative time points.
Deng et al. [59]	2022	Retrospective	N	VAS scores were independently filled in by patients after the doctor's brief explanation, with 0 being no pain and 10 being very painful.
Molina et al. [60]	2023	Prospective randomized control	N	Preoperative data, including ODI, SF-36, lumbar and lower extremity VAS, BMI, hematocrit, and temperature, were recorded.
Luan et al. [61]	2022	Retrospective	N	Functional improvement was assessed by VAS for lumbar and lower extremity pain, Japanese Orthopaedic Association, and ODI before surgery, after surgery, and at the last follow-up.
Tian et al. [62]	2023	Retrospective	N	The preliminary effect was evaluated by VAS, ODI, and modified Macnab scale at preoperative, postoperative 1 month, and the last follow-up.
Zhang et al. [63]	2022	Retrospective	N	The VAS, the ODI, and laboratory tests, including preoperative and postoperative erythrocyte sedimentation rate and C-reactive protein, were recorded.
Levy et al. [64]	2023	Retrospective	N	Preoperative and postoperative office visits took place at orthopedic clinical sites in the city proper and greater metropolitan area as dictated by patient preference and surgeon availability.
Nie et al. [65]	2023	Retrospective	N	PROMs for physical function, mental function, disability, and pain were collected at preoperative and postoperative 6-week, 12-week, 6-month, and 1-year time points.
Nie et al. [66]	2023	Retrospective	N	PROMs for physical function, anxiety, pain interference, sleep disturbance, depression, back pain, leg pain, and disability outcomes were recorded at preoperative and postoperative 6-week, 12-week, 6-month, 1-year, and 2-year time points.
Patel et al. [67]	2022	Retrospective	N	PROMs, including Patient-Reported Outcomes Measurement Information System Physical Function (PROMIS-PF), 12-Item Short Form Physical Composite Score, VAS back, VAS leg, and ODI were collected at preoperative, 6-week, 12-week, 6-month, 1-year, and 2-year time points.
Toci et al. [68]	2023	Retrospective	N	PROMs were collected through our institution’s prospectively managed outcomes database.
Jia et al. [69]	2022	Retrospective	N	VAS is one of the commonly used pain scoring criteria, which divides the degree of pain from 0 to 10: 1–3 is mild pain, 4–6 is moderate pain, and 7–10 is severe pain.
Liu et al. [70]	2023	Prospective case control	N	The VAS score for low back and leg pain was evaluated using a questionnaire containing a 10-cm line with “none” on one end of the scale and “severe pain” (10) on the other end.
Zou et al. [71]	2022	Prospective cohort	N	These outcomes were assessed preoperatively and at 3 months, 6 months, and 12 months postoperatively.
Lainé et al. [72]	2022	Retrospective	N	Patients were clinically evaluated before surgery and during follow-up. Patients were also asked to fill out questionnaires (Visual Analogue Scale (VAS) for lumbar and radicular pain, ODI, and Scoliosis Research Society-22 (SRS-22) score) and to assess their walking distance.
Song et al. [73]	2022	Retrospective	N	In our study, the VAS back pain score and leg pain were used to assess back pain preoperatively and at the last follow-up.
Jacob et al. [74]	2022	Retrospective	N	PROMs were collected at preoperative, 6-week, 12-week, 6-month, 1-year, and 2-year postoperative time points.
Sheng et al. [75]	2022	Retrospective	N	The VAS pain score significantly decreased from preoperative 6.9 ± 1.1 to 1.3 ± 0.7 3 months after operation.
Lambrechts et al. [76]	2023	Retrospective	N	Secondary outcomes included preoperative and 3-month or 1-year postoperative PROMs.
Aoki et al. [77]	2022	Retrospective	N	Clinical scores were evaluated preoperatively and at 5 years postoperatively.
Gao et al. [78]	2022	Retrospective	N	The clinical outcomes were measured using the VAS of LBP and lower extremity pain.
Jacob et al. [79]	2022	Retrospective	N	Outcome measures were all collected at a baseline preoperative time point.
Zhang et al. [80]	2022	Retrospective	N	The VAS was 7.1 ± 0.7 in group A and 7.2 ± 0.6 in group B pre-operatively.
Chen et al. [81]	2022	Retrospective	N	VAS is one of the commonly used pain scoring criteria, which divides the degree of pain from 0 to 10: 1–3 is mild pain, 4–6 is moderate pain, and 7–10 is severe pain.

## Discussion

Our study performed a retrospective analysis to assess the degree of variability in self-reported pain between preoperative clinic visits and DOS in patients having procedures associated with preoperative pain. The primary analysis was to determine if there were differences in NRS pain scores at the clinic and the DOS in patients undergoing TKA, THA, and spinal fusion, which are procedures associated with significant preoperative pain [82-84]. Our results revealed a significant 3-point decrease in self-reported pain between these visits. When stratified by surgical indication or gender, comparable differences between clinic and DOS NRS scores were found for male and female patients, as well as those scheduled for TKA and THA surgery. Interestingly, we did find that the magnitude of the difference was lower for spinal fusion than for TKA and THA. Furthermore, temporal analysis of the timing of the clinic visit found no difference in NRS scores between one and ninety days prior to surgery. To our knowledge, this is the first report to reveal significant variability in preoperative pain scores based on the assessment time and location.

This study raises several important and unresolved questions. First and foremost, what are the factor(s) that induce differential self-reporting of pain scores between the clinic and DOS? There are clear differences between these settings, including factors involving the team member providing the pain assessment, distant vs. impending surgery, patient expectations, time of day, fasting, and hydration. Surprisingly, little research has been published on the influence of such factors on patient-reported pain. Martin et al. evaluated self-reported pain in foot and ankle orthopedic patients and found that ~80% of patients reported higher pain scores to the surgeon compared to nurses, with the magnitude of this difference averaging 2.9 points [85]. Meyer-Friessem et al. examined postoperative pain in cardiac surgery patients and noted that patients reported more intense pain to female compared to male assessors [86]. Others have also reported on psychological factors that impact pain, with anxiety and catastrophizing both shown to increase both pre- and postoperative self-reported pain [87,88]. However, it is not known how these types of factors may change from the clinic to the DOS. Another possible psychological factor contributing to our findings is the anticipation of pain resolution. Given the utility of preoperative pain as a predictor of postoperative pain, future studies will be necessary to determine whether pain scores obtained in the clinic or DOS exhibit greater predictive power and to identify the precise factors, such as patient psychological characteristics (e.g., anxiety, catastrophizing, anticipation) or institutional characteristics (e.g., position/gender of rater, location) that contribute to the differences in pain.

Our qualitative literature review found that details of the time and place of preoperative pain score collection are rarely reported in the literature. We reviewed 24 TKA studies, and only the study by Meena et al. provided clear details about pain score collection [16]. All others were vague in their description the preoperative time and location, such as Ao et al. [17], who stated, “The pain was measured by the visual analog scale (VAS) pre- and postoperatively” and Kenanidis et al. [18], who stated, “Complications, visual analogue scale score (VAS), and Oxford Knee Score (OKS) were assessed preoperatively and for six postoperative months.” Similarly, for the THA studies, out of 20 studies, only the one by Singh et al. reported preoperative pain score collection in a clear manner [44]. Others were unclear, as evidenced by Kobayashi et al. [43], who reported, “The visual analog scale (VAS) was used to assess knee pain severity before and at three months after THA,” and Agerholm et al. [49], who reported that, “Data are based on patients self-reported data from both pre- and post-operative surveys as well as medical data registered by health care staff.” Finally, for spinal fusion, all of the 25 reviewed studies were unclear as to the time of preoperative pain score collection, with Deng at al. [59] stating, “VAS scores were independently filled in by patients after the doctor's brief explanation, with 0 being no pain and 10 being very pain,” and Nie et al. [65] stating, “PROMs for physical function, mental function, disability, and pain were collected at preoperative and postoperative six weeks, 12 weeks, six months, and one year time points.” As a whole, this review indicates that the description of the time and location of preoperative pain scores in the literature is too inconsistent to allow for meaningful comparison from study to study. 

Limitations and strengths of the study

Our study has several limitations. This was a retrospective study of pain scores collected as part of routine clinical care, with pain questionnaires administered by multiple treatment teams. This means that some patients reported pain on paper forms, while others were asked about their pain by providers ranging from nurses to physician assistants to orthopaedic surgeons. Our statistical analyses also entailed several subgroup analyses, and these had less power than the primary analysis. Another limitation is that this study was conducted at a single suburban academic institution in the Northeast United States. Finally, this study was not designed to determine which pain scores more accurately reflect patient pain. This would require a prospective study with well-controlled pain assessments, which were not possible in this retrospective analysis.

However, these limitations are balanced by several important strengths. First, this study had a large sample size (n=1,027) in the primary analysis population. Furthermore, our study design included a group of patients expected to have minimal pain at both study time points. As expected, this other group reported minimal pain, which strengthens the internal validity of our study since it supports the accuracy of pain scores recorded in both the clinic and on DOS. Other strengths of the study include standardization of data collection and regular audits to ensure consistency.

## Conclusions

Our retrospective analysis identified a significant difference in self-reported pain between clinic visits and DOS for patients scheduled to undergo procedures associated with preexisting pain. These results highlight the importance of defining the setting where pain measurements are conducted, which is supported by our qualitative analysis demonstrating that the time and location of pain assessments are rarely reported. Furthermore, assessor characteristics (e.g., gender, position) have also been shown to impact self-reported pain. Therefore, we recommend that future studies that include preoperative pain as an outcome measure report the time, location, and manner of pain score collection, as well as assessor characteristics, to reduce variability and facilitate uniform comparisons between studies.
